# Molecular Analysis of *Aedes aegypti* Classical Protein Tyrosine Phosphatases Uncovers an Ortholog of Mammalian PTP-1B Implicated in the Control of Egg Production in Mosquitoes

**DOI:** 10.1371/journal.pone.0104878

**Published:** 2014-08-19

**Authors:** Debora Monteiro Moretti, Lalima Gagan Ahuja, Rodrigo Dutra Nunes, Cecília Oliveira Cudischevitch, Carlos Renato Oliveira Daumas-Filho, Priscilla Medeiros-Castro, Guilherme Ventura-Martins, Willy Jablonka, Felipe Gazos-Lopes, Raquel Senna, Marcos Henrique Ferreira Sorgine, Klaus Hartfelder, Margareth Capurro, Georgia Correa Atella, Rafael Dias Mesquita, Mário Alberto Cardoso Silva-Neto

**Affiliations:** 1 Laboratório de Sinalização Celular (LabSiCel), Instituto de Bioquímica Médica Leopoldo de Meis, Universidade Federal do Rio de Janeiro, Rio de Janeiro, RJ, Brazil; 2 Department of Pharmacology, University of California San Diego, San Diego, California, United States of America; 3 Departamento de Biologia Celular e Molecular e Bioagentes Patogênicos, Faculdade de Medicina de Ribeirão Preto, Universidade de São Paulo, Ribeirão Preto, Brazil; 4 Departamento de Parasitologia, Instituto de Ciências Biomédicas, Universidade de São Paulo, São Paulo, Brazil; 5 Departamento de Bioquímica, Instituto de Química, Universidade Federal do Rio de Janeiro, Rio de Janeiro, RJ, Brazil; 6 Instituto Nacional de Ciência e Tecnologia em Entomologia Molecular (INCT-EM), Rio de Janeiro, RJ, Brazil; Centro de Pesquisas René Rachou, Brazil

## Abstract

**Background:**

Protein Tyrosine Phosphatases (PTPs) are enzymes that catalyze phosphotyrosine dephosphorylation and modulate cell differentiation, growth and metabolism. In mammals, PTPs play a key role in the modulation of canonical pathways involved in metabolism and immunity. PTP1B is the prototype member of classical PTPs and a major target for treating human diseases, such as cancer, obesity and diabetes. These signaling enzymes are, hence, targets of a wide array of inhibitors. Anautogenous mosquitoes rely on blood meals to lay eggs and are vectors of the most prevalent human diseases. Identifying the mosquito ortholog of PTP1B and determining its involvement in egg production is, therefore, important in the search for a novel and crucial target for vector control.

**Methodology/Principal Findings:**

We conducted an analysis to identify the ortholog of mammalian PTP1B in the *Aedes aegypti* genome. We identified eight genes coding for classical PTPs. *In silico* structural and functional analyses of proteins coded by such genes revealed that four of these code for catalytically active enzymes. Among the four genes coding for active PTPs, AAEL001919 exhibits the greatest degree of homology with the mammalian PTP1B. Next, we evaluated the role of this enzyme in egg formation. Blood feeding largely affects AAEL001919 expression, especially in the fat body and ovaries. These tissues are critically involved in the synthesis and storage of vitellogenin, the major yolk protein. Including the classical PTP inhibitor sodium orthovanadate or the PTP substrate DiFMUP in the blood meal decreased vitellogenin synthesis and egg production. Similarly, silencing AAEL001919 using RNA interference (RNAi) assays resulted in 30% suppression of egg production.

**Conclusions/Significance:**

The data reported herein implicate, for the first time, a gene that codes for a classical PTP in mosquito egg formation. These findings raise the possibility that this class of enzymes may be used as novel targets to block egg formation in mosquitoes.

## Introduction

Tyrosine phosphorylation is part of a complex cell repertoire that first appeared nearly 600-million years ago and is largely responsible for the emergence of the first multicellular animals [1]. Protein tyrosine phosphatases (PTPs) are enzymes that catalyze tyrosine dephosphorylation and regulate central steps of cell biology. The PTP family is composed of four different subfamilies. The active sites of classes I, II and III each harbor a cysteine, which is involved in catalysis. In Class IV, this cysteine is replaced by aspartic acid [2]. Class I Cys-PTPs are the largest group of PTPs and are divided into “classical” and dual specificity phosphatases. Classical phosphatases are enzymes that are strictly devoted to the dephosphorylation of phosphotyrosine residues. Classical PTPs have been further subdivided into receptor PTPs and soluble or non-transmembrane PTPs [3].

*Aedes aegypti* is the vector of Dengue and yellow fever. Once it feeds on blood, a complex series of signaling events lead to yolk synthesis and egg formation. Synthesized by the female mosquito fat body, vitellogenin (Vg), the main yolk protein, is the ultimate result of a chain of endocrine and signaling events that are still not completely understood. It has been shown that, after a blood meal, the amino-acid concentration in the hemolymph increases and the synthesis of brain-derived signaling molecules, such as insulin-like peptides, is induced [4]–[6]. Such peptides stimulate the ovaries to produce ecdysone, which then induces the fat body to produce Vg. Vg production by the fat body also relies on amino acids derived from blood digestion, which activate the TOR/S6k signaling cascade [7], [8]. Vg is then secreted by the fat body into the hemolymph and taken up by the developing follicles via receptor-mediated endocytosis. In mosquitoes, the interaction of insulin or insulin-like peptides with the mosquito insulin receptor (MIR) triggers the PI3K/Akt pathway and promotes the production of ecdysteroids, the regulation of egg formation and immunity [9], [10]. Furthermore, inhibition of PTPs an antagonist of the insulin pathway decreases ecdysteroid production by mosquito ovaries [9]. Silencing of the Phosphatase and Tensin homologue (PTEN), an antagonist of the PI3K pathway, leads to an increase in egg formation [10]. The above studies suggest the presence of PTPs as modulators of egg formation in mosquitoes, but the genes coding for these enzymes have not yet been identified. It is possible that the inhibition of PTP activity encoded by such genes may ultimately reduce or impair the ability of female mosquitoes to lay eggs, as demonstrated for other components involved in vitellogenesis [11], [12].

In the study reported herein, we conducted a bioinformatics analysis of the *A. aegypti* genome to identify the mosquito ortholog of PTP1B and determine its involvement in egg formation [13]. The inhibition of these regulators or the blocking of proteins under their transcriptional control can potentially provide new targets for suppression of egg formation and pathogen transmission by mosquitoes [14]. Among the genes that encode mosquito PTPs, AAEL001919 has the highest (53%) identity with human PTP1B. The treatment of blood-fed mosquitoes with classical PTP inhibitors or the silencing of this gene through RNAi partially blocked egg production. Thus, AAEL001919 may present a potential target for the control of tyrosine phosphorylation in mosquitoes and may ultimately be used to decrease mosquito reproduction.

## Results

### *In silico* analysis of soluble PTP sequences

**Figure 1:**
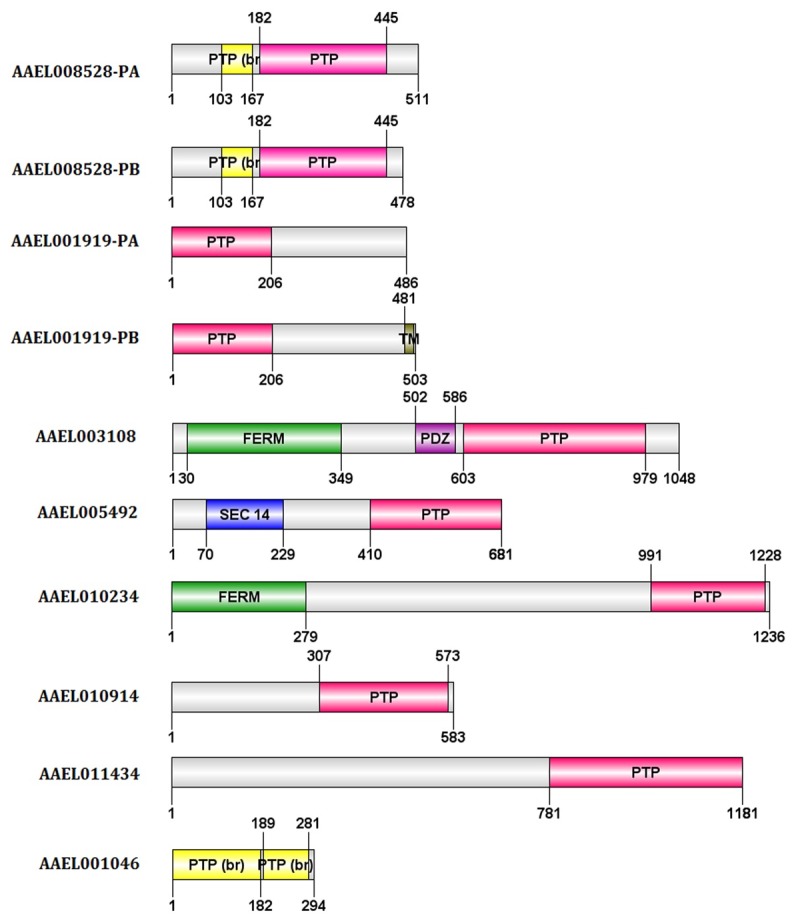
Domain architecture of *A. aegypti* Protein Tyrosine Phosphatases. The ten Protein Tyrosine Phosphatase sequences from *A. aegypti* were analyzed for the presence biologically active domains. The domains were identified using a conserved sequence search as mentioned in the methods. Domain architecture was mapped onto the sequence using the DOG 1.0 protein-structure illustrator (DOG 1.0: illustrator of protein domain structures). Nomenclature used is as follows: PTP - Protein Tyrosine Phosphatase domain, PTP (br) - Absent Protein Tyrosine Phosphatase domain, SEC14 - Phosphatidylinositol transfer protein domain, FERM - F for 4.1 protein, E for ezrin, R for radixin and M for moesin, cytoskeletal association domain (includes the FA: FERM-associated domain) PDZ - also called DHR or GLGF, adaptor domain.

The search for PTP sequences in the *Aedes aegypti* genome has led to the discovery of 8 PTP genes coding for 10 proteins belonging to Class I soluble PTPs: AAEL001046, AAEL001919-PA, AAEL001919-PB, AAEL003108, AAEL005492, AAEL008528-PA, AAEL008528-PB, AAEL010234, AAEL010914 and AAEL011434 (Figure 1). Among the genes that code for classical PTPs in *Aedes*, two, AAEEL001919 and AAEL008528, presented mRNA splice variants denoted as RA or RB. For each of these genes, the distinguishing character was the presence of at least one classical PTP domain (Figure 1). Some PTPs showed accessory domains, including AAEL003108, that harbored adaptor domains, including FERM and PDZ, which are crucial for signal transduction (Figure 1). Several of these sequences also showed the presence of inactive or absent PTP domains, the significance of which can be linked to the role of pseudo-phosphatases in signaling. Therefore, these sequences may function as adaptor molecules that link pathways rather than fill catalytic roles [15].

### Analysis of PTP sequences and their specific motifs

As the PTP domain is defined by ten sequence and structural motifs [16], the sequences of interest were analyzed in detail for PTP motifs. Of the ten motifs, special attention was given to the active-site motifs, motifs 1, 8, 9 and 10. The other six motifs (2, 3, 4, 5, 6, 7) form the core of the PTP protein and are implicated in the thermodynamic stabilities of the molecules. Sequence conservation in the four active-site motifs was, therefore, used as a tool to classify the PTP sequences into active or inactive PTP domains (Table 1 and Table 2). PTP activity is critically dependent on the nucleophilic cysteine present in Motif 9 (also called the HCS motif) [17], [18]. Motif 1 (also called the KNRY loop), while not directly involved in the reaction per se, is crucial for recruiting the phosphotyrosine into the PTP active site and distinguishes PTPs from serine/threonine phosphatases [19].

**Table 1:** Analysis of PTP catalytic sequences.

Protein Accession number	Number of PTP domains	PTP domain Boundary (amino acid sequence number)	Sequence of Motif 1, Motif 8, Motif 9 and Motif 10	Motifs active/inactive	PTP domain predicted active/inactive
AAEL001046	2	PTP ABSENT (1–182)	INACTIVE/DOMAIN ABSENT	INACTIVE	INACTIVE
		PTP ABSENT (189–281)	INACTIVE/DOMAIN ABSENT	INACTIVE	INACTIVE
AAEL001919-PA	1	1–206	(ABSENT) YTTWPDFGIP PIIHCSAGIGRSGT IQTVDQLYF	INACTIVE ACTIVE ACTIVE ACTIVE	ACTIVE
AAEL001919-PB	1	1–206	(ABSENT) YTTWPDFGIP PIIHCSAGIGRSGT IQTVDQLYF	INACTIVE ACTIVE ACTIVE ACTIVE	ACTIVE
AAEL003108	1	603–979	NLNKNRY YLAWPDHGVP PIIHCSAGIGRTG VQNVSQYRF	ACTIVE ACTIVE ACTIVE ACTIVE	ACTIVE
AAEL005492	1	410–681	NLAKNRY FTSWPDYGVP PMVVHCSAGIGRT IQMPDQYVF	ACTIVE ACTIVE ACTIVE ACTIVE	ACTIVE
AAEL008528-PA	2	PTP (ABSENT) 103–167	INACTIVE/DOMAIN ABSENT	INACTIVE	INACTIVE
		PTP (205–445)	NESKHKR FQVWPDHGVP PICVHCSAGIGRT VQTEAQYKF	INACTIVE ACTIVE ACTIVE ACTIVE	ACTIVE
AAEL008528-PB	2	PTP (ABSENT) 103–167	INACTIVE/DOMAIN ABSENT	INACTIVE	INACTIVE
		PTP (205–445)	NESKHKR FQVWPDHGVP PICVHCSAGIGRT VQTEAQYKF	INACTIVE ACTIVE ACTIVE ACTIVE	ACTIVE
AAEL010234	1	991–1228	NKARNF YNEWGDQNCP PPVLIHCNEGGGRTPSLAQYKF	ACTIVE INACTIVE INACTIVE INACTIVE	INACTIVE
AAEL010914	1	307–573	KNRSID LWPKQSA NCLNGSDRSC DPNHMQL	INACTIVE INACTIVE INACTIVE INACTIVE	INACTIVE
AAEL011434	1	781–1181	QSKNRY FPDWPDHRSP PIIHCSAGIGRTG VQNSEQYEL	ACTIVE ACTIVE ACTIVE ACTIVE	ACTIVE

**Table 2:** Analysis of the superposition Mosquito PTPs with human PTP1B.

Protein	Amino Acids Modeled	Template PTP structure Name/PDB ID	Percentage Sequence Identity	Root Mean Square Deviation (RMSD) from PTP1B structure (2AZR)(C-α RMSD) (Å)
AAEL001919	229	TCPTP (2I1Y)	34%	0.283
AAEL005492	299	MEG2/PTPN9(2PA5)	47%	1.546
AAEL011434	283	PCPTP1/PTPR (2A8B)	29%	2.323
AAEL003108	262	PTPH1/PTPN3(2B49)	46%	1.510
AAEL008528	309 (PTP domain)	SHP2/PTPN11 (3B7O)	60%	1.1412
	188 (Absent PTP domain)	CD45 (1YGU)	19%	---

**Figure 2:**
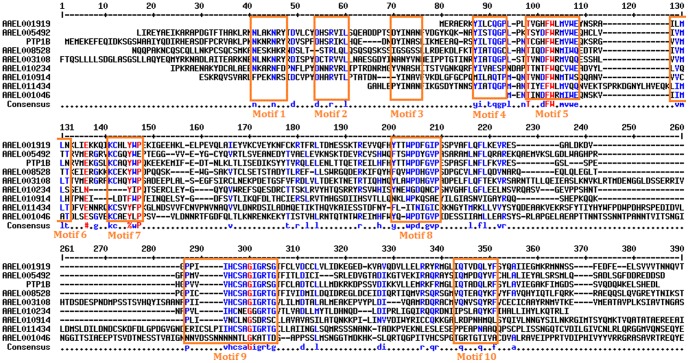
Multiple sequence alignments of the *A. aegypti* PTP domains with the PTP domain of human PTP1B. Regions of high sequence similarity (>90\%) are highlighted in red; regions of moderate sequence identity (50–90%) are highlighted in blue. The ten motifs defining a PTP domain are marked in red boxes. These are in accordance with the nomenclature of Andersen *et al.* [59].

Of the ten PTP sequences analyzed, the following seven showed the presence of at least one active PTP domain: AAEL001919-PA, AAEL001919-PB, AAEL005492, AAEL011434, AAEL008528-PA, AAEL008528-PB and AAEL003108 (Table 1 and Figure 2). The active domains of the splicing variants of AAEL001919 and AAEL008528 were identical (Table 1 and Figure 2). A multiple sequence alignment of these sequences with human PTP1B showed the presence of all ten conserved PTP motifs (Figures 1 and 4). The sequences of these proteins were subsequently used to obtain molecular models to enable further study of their structure-function relationships.

### Structural analysis of mosquito PTP domains

**Figure 3:**
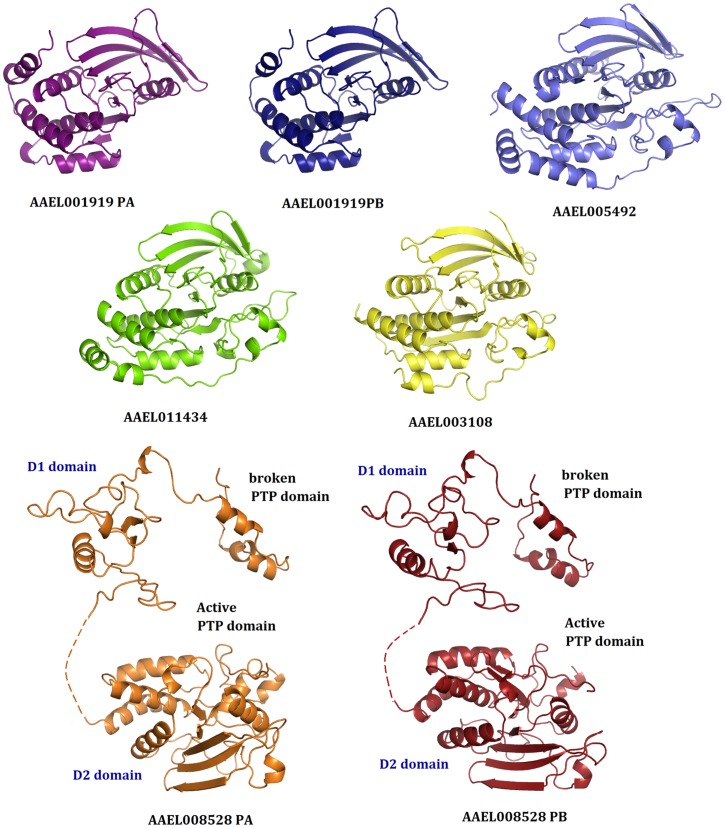
Structural models of the ‘active’ PTP sequences from *A. aegypti*. All proteins showed the classical PTP fold with a twisted β sheet at the center surrounded by α helices. Because of its low sequence conservation, the absent PTP domain of AAEL008528 could not be modelled completely. Only one model is shown for genes with splicing variants because their PTP domains are identical. For additional details, please check Figure 4.

**Figure 4:**
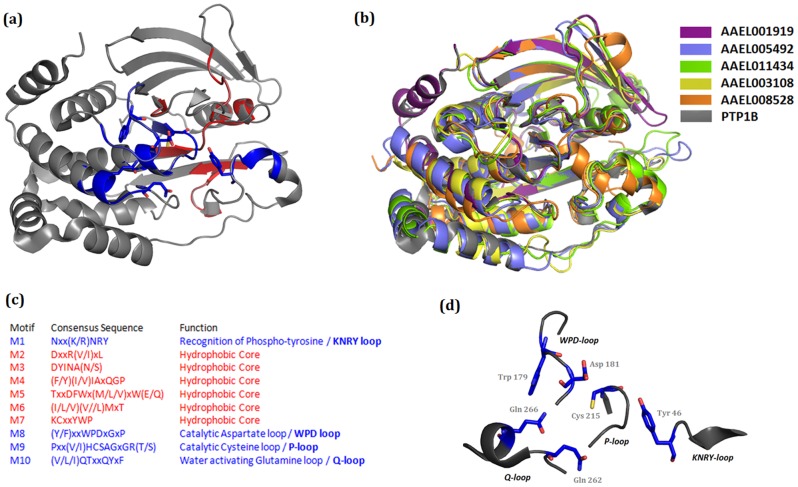
Superimposition of *A. aegypti* ‘active’ PTP model structures with PTP1B as the reference. (A) The classical PTP-domain as seen in PTP1B (PDB ID: 2AZR). The ten motifs defining the conserved PTP domain are highlighted (B) Superimposition of the active PTP-domains of *Aedes agypti* onto the structure of PTP1B (C) Sequence conservation defining the conserved motifs of the PTP domain. Four motifs viz., M1, M8, M9 and M10 harbour the active site residues of the PTP domain. The sequence of these motifs as seen in the *Aedes aypti* PTP domains are mentioned in Table 1. (D) Active site of the PTP-domain as seen in PTP1B.

Molecular models of the five active PTP domains from the above seven different proteins were obtained using the previously solved homologous PTP-1B structure as a template (Figure 3, Table 1). Each of the structures showed the classical PTP fold with a twisted β-sheet composed of eight β-strands at the center flanked by eight α-helices. The active-site cysteine was observed at the center of the active site flanked by the general acid aspartate from Motif 8. Motif 10 glutamines were also observed at the active site (Figure 4). A superposition of these structures with human PTP1B showed that the geometry of the central active site is extremely conserved (Figure 4 and Table 2). This result is consistent with the information obtained from multiple sequence alignments which showed that the ten motifs to be extremely conserved (Figure 2).

### Evolutionary analysis of mosquito PTP sequences

**Figure 5:**
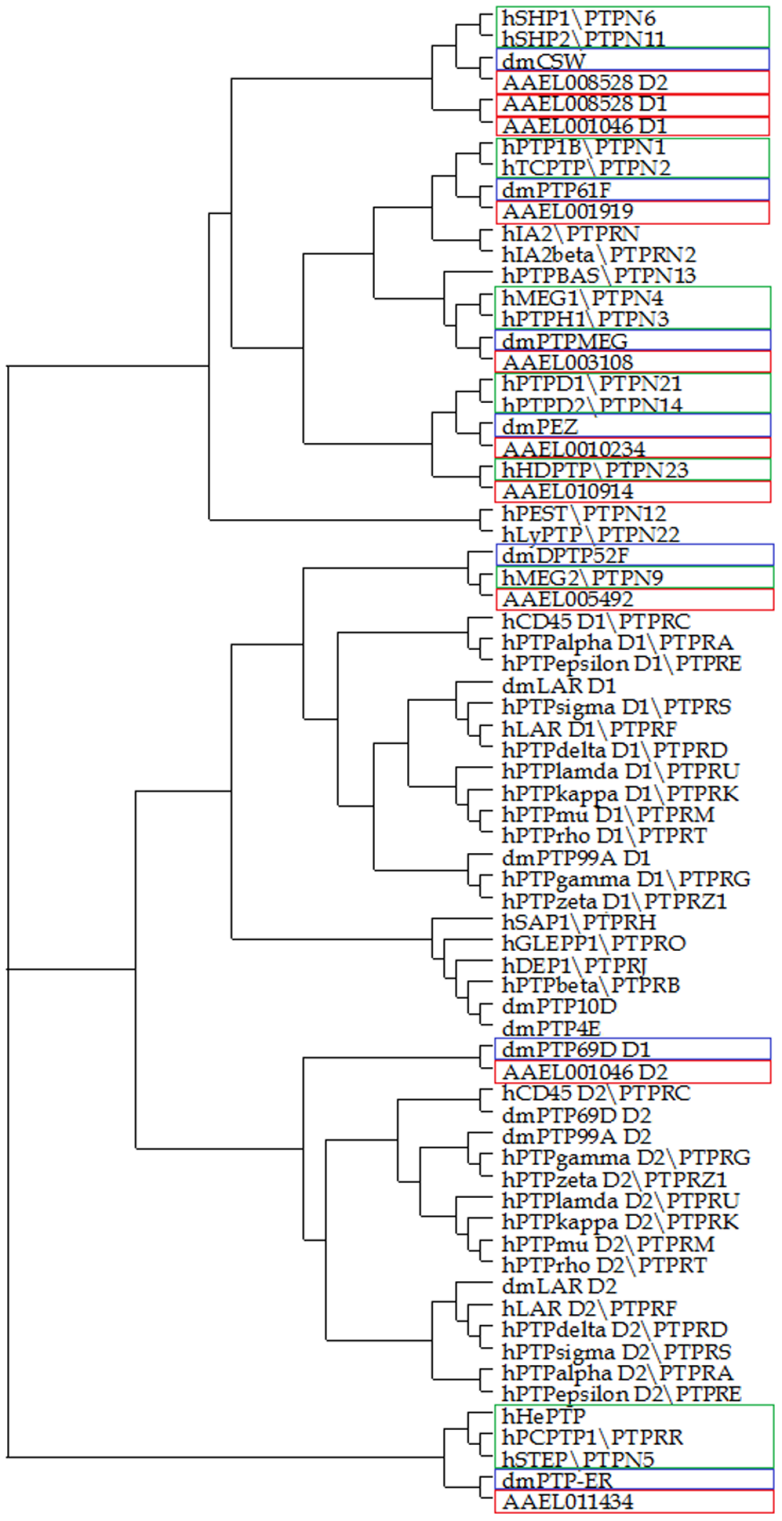
Evolutionary analysis of *Aedes* PTP sequences with the well-characterized PTPs from *H. sapiens* and *D. melanogaster*. PTP domains from *A. aegypti* and from sequences derived from human and fly (names starting with “h” and “dm”) were clustered genomes. The *A. aegypti* active PTP domains are highlighted in red, while the closest human and fly PTPs are highlighted in blue and green, respectively.

A phylogram of the *A. aegypti* PTP domains was made using the previously characterized PTPs of humans (*Homo sapiens*) and flies (*Drosophila melanogaster*) (Figure 5). These genomes were chosen because of their closeness with the mosquito genome (*D. melanogaster*) and for their extensive biochemical and structural characterizations (*H. sapiens*). The phylogram was generated using a multiple sequence alignment at the CLUSTALW web-server. The neighbor-joining algorithm was used to obtain the evolutionary tree using the TREEVIEW software package [20]. From the phylogram, it was evident that both active and inactive *A. aegypti* PTP domains were closely related to the vertebrate and fly PTP domains. The well-formed PTP domain of AAEL008528 was observed to be closest to the *D. melanogaster* Corkscrew PTP. Belonging to the SHP1 family, these PTPs are characterized by the presence of SH2 in addition to their tyrosine phosphatase domains. Interestingly, the absent PTP domain of AAEL008528 also clustered near these proteins (along with the absent N-terminal PTP domain of AAEL001046), indicating a plausible role for these absent PTP domains as adapter modules. The single PTP domains AAEL05492, AAEL003108 and AAEL001919 clustered separately into three different groups, which were closest to the *D. melanogaster* DPTP52F, dm PTPMEG, dmPTP61F and *H. sapiens* MAG2, MEG1, PTP1B, respectively. The phylogram also indicated that the inactive and active PTPs from *A. aegypti* clustered separately, but the proximity of the inactive PTP sequences to the other PTP homologues indicates that these could function as signaling molecules, much like adaptor proteins. The gene AAEL011434 was also described in the analysis conducted by Hatzihristidis et al. 2013 [21]. However, in *Drosophila*, this enzyme is a negative modulator of the MAPK pathway and is, thus, not considered in this study as a true member of the classical PTP group [22].

### PTP involvement in mosquito egg formation

**Figure 6:**
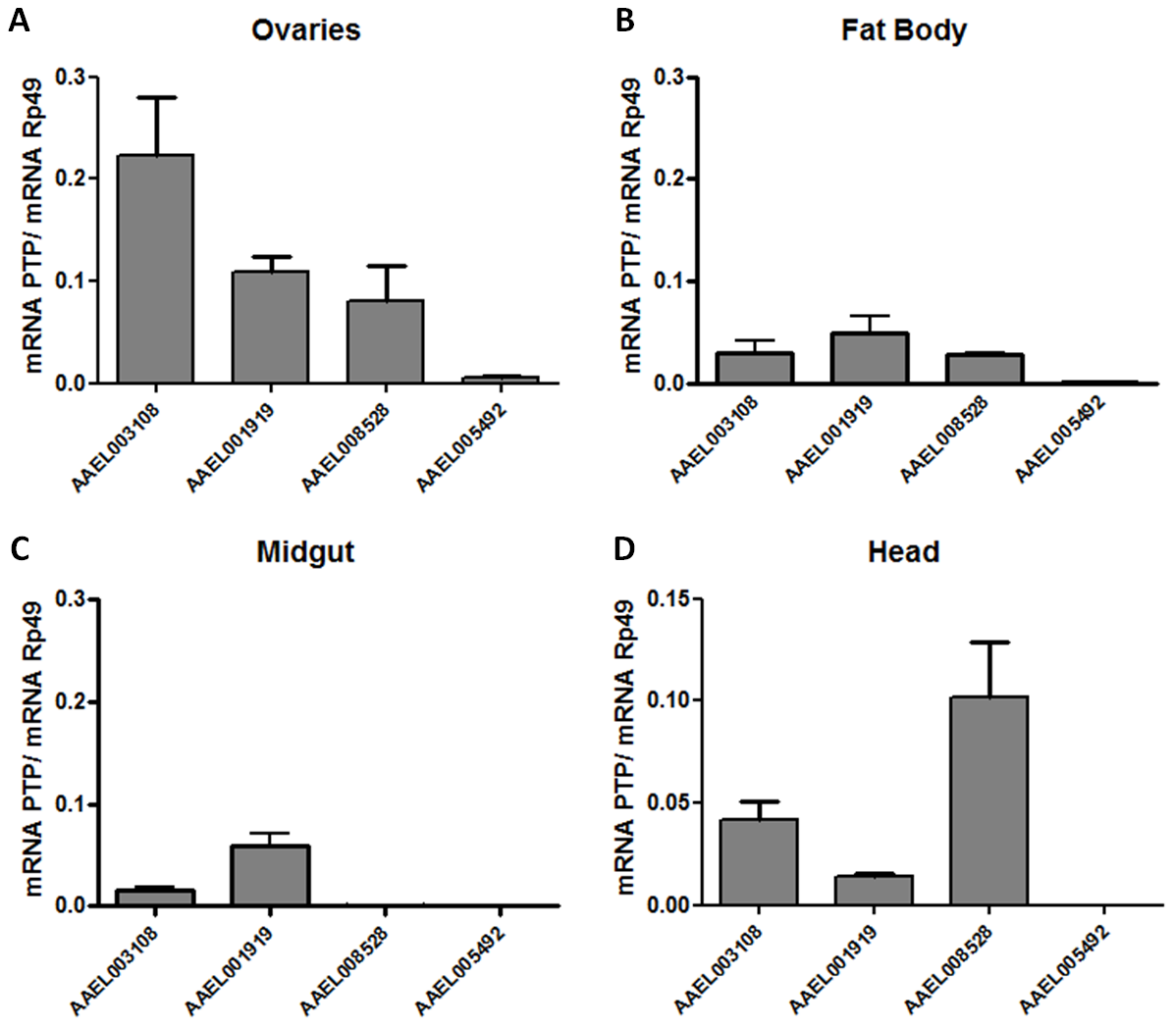
Expression levels of classical PTPs in *Aedes* tissues. Female mosquitoes (20–30 insects) were maintained on 10% sucrose *ad libitum* and were collected at four and seven days after emergence from the pupal stage. They were dissected, and RNA was extracted from different tissues. Expression levels of PTPs were measured using qPCR. The following tissues were evaluated: (A) Ovaries; (B) Fat body; (C) Midgut and (D) Head. Expression levels of the following genes were evaluated: AAEL003108, AAEL001919, AAEL008528, AAEL005492. The latter were normalized against the expression of the mosquito Rp49 gene. Data are the means ± S.E.M of three different experiments.

The involvement of a complex set of intracellular signaling pathways in the regulation of Vg synthesis and egg formation has been described in the literature [23]. To determine the involvement of PTPs in mosquito reproduction, we initially evaluated the expression in different tissues of the 4 PTP genes that code for active enzymes (AAEL005492, AAEL008528, AAEL003108, and AAEL001919). AAEL011434 was not analyzed because this gene was identified as a PTP after the major experiments were completed and because it seems to be a dual-specificity phosphatase that modulates MAPKs [22]. AAEL003108 and AAEL001919 are the only PTPs detectable in all tissues isolated from sucrose-fed mosquitoes (Figure 6). In contrast, AAEL005492 had very low concentration in all tissues. Curiously, AAEL008528 is significantly expressed in the mosquito head, concomitant with a transient increase in phosphotyrosine phosphorylation that may involve fluctuations in the expression levels of AAEL008528 [24]. AAEL008528 is orthologous to *Drosophila* corkscrew, a PTP that was discovered in mutations related to defects in embryonic development [25], [26]. AAEL003108 clusters with the *Drosophila* dmPTPMEG. PTPMEG is a cytoplasmic PTP containing FERM (F for 4.1 protein, E for ezrin, R for radixin and M for moesin) and PDZ (P for postsynaptic density protein (PSD95)), D for *Drosophila* disc large-tumor suppressor (Dlg1), and Z for zonula occludens-1 protein (zo-1)) domains. AAEL001919 clusters with the *Drosophila* PTP dmPTP61F [29], [30], [31]. In mammalian T cells, PTP (TCPTP) and PTP1B share a high level of structural similarity but display different functions, which are defined through the presence of specific non-catalytic domains that alter enzyme localization within the cell.

AAEL003108 clusters with the *Drosophila* dmPTPMEG. PTPMEG is a cytoplasmic PTP containing FERM (F for 4.1 protein, E for ezrin, R for radixin and M for moesin) and PDZ (P for postsynaptic density protein (PSD95)), D for *Drosophila* disc large-tumor suppressor (Dlg1), and Z for zonula occludens-1 protein (zo-1)) domains. In *Drosophila*, the vertebrate homologs of PTPN3 (PTPH1) and PTPN4 (MEG1) are expressed in the nervous system, where they are involved in the regulation of axon projections [27], [28]. PTP 003108 was not further addressed in the following experiments no matter it is clearly more expressed in the ovaries due to two reasons: its lack of function in the mammalian models and its lack of effect on early silencing experiments of egg formation and [28]

AAEL001919 clusters with the *Drosophila* PTP dmPTP61F [29], [30], [31]. In mammalian T cells, PTP (TCPTP) and PTP1B share a high level of structural similarity but display different functions, which are defined through the presence of specific non-catalytic domains that alter enzyme localization within the cell. PTP activity against pNPP using tissues from sucrose-fed mosquitoes is highly sensitive to sodium orthovanadate (data not shown). In addition, in our pNPP assays with mosquito tissues, the inclusion of a peptide that contains a phosphotyrosine dephosphorylated by the mammalian PTP1B leads to a 40–90% inhibition of total PTP activity (Figure S1), indicating the presence of catalytically active mosquito enzymes possessing the same biochemical properties as mammalian PTPs.

**Figure 7:**
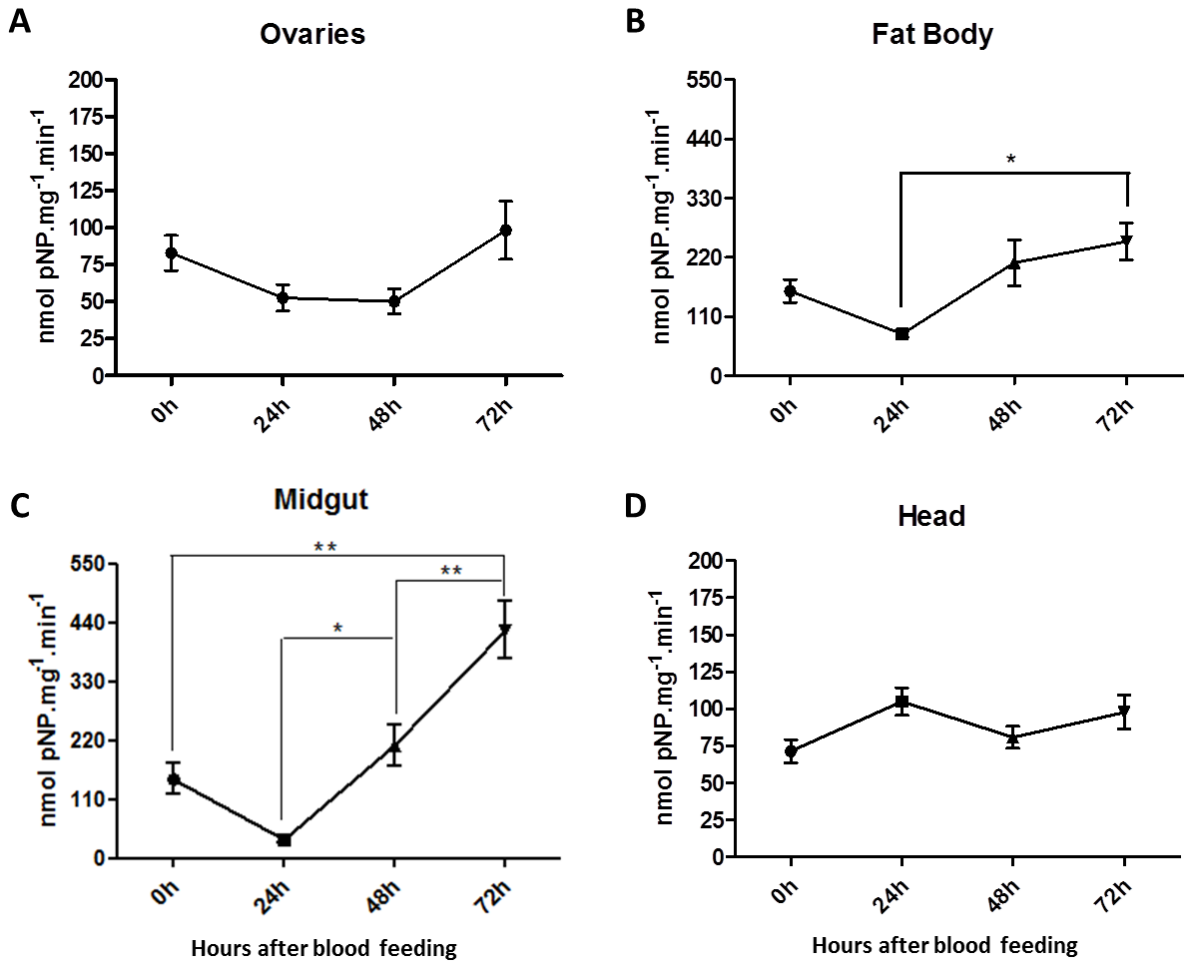
PTP activity in mosquito tissues after blood feeding. Five- and seven-day old female mosquitoes (20–30 insects) were fed blood on rabbit skin were dissected 24 h, 48 h and 72 h later. Tissues were assayed for PTP activity using pNPP as the substrate. The following tissues were evaluated: (A) ovary; (B) fat body; (C) midgut and (D) head. The control specimens (0 h) were maintained in 10% sucrose *ad libitum* and were dissected with one of the three points. Normalized data from five experiments were analyzed by one-way analysis of variance (ANOVA) and by a post-test Tukey's Multiple Comparison Test, p<0.05 (A) *p = 0.0476*, (B) *p = 0.0293*, (C) *p = 0.0001*, (D) *p = 0.1494*.

The above overall analysis suggests that AAEL001919 is likely to be the mosquito ortholog of mammalian PTP1B and *Drosophila* dmPTP61F. To determine the role of AAEL001919 in egg formation, we performed several experiments. Because *Aedes* is an anautogenous mosquito, we evaluated the impact of blood feeding on aspects of PTP function. Initially, we evaluated the effect of blood feeding on total PTP activity using pNPP as substrate (Figure 7). Blood feeding induced a decrease in total enzyme activity at 24 hours in all tissues except the mosquito head. Major changes in PTP activity were detected in both the mosquito midgut and the fat body during the first 72 hours following the blood meal.

**Figure 8:**
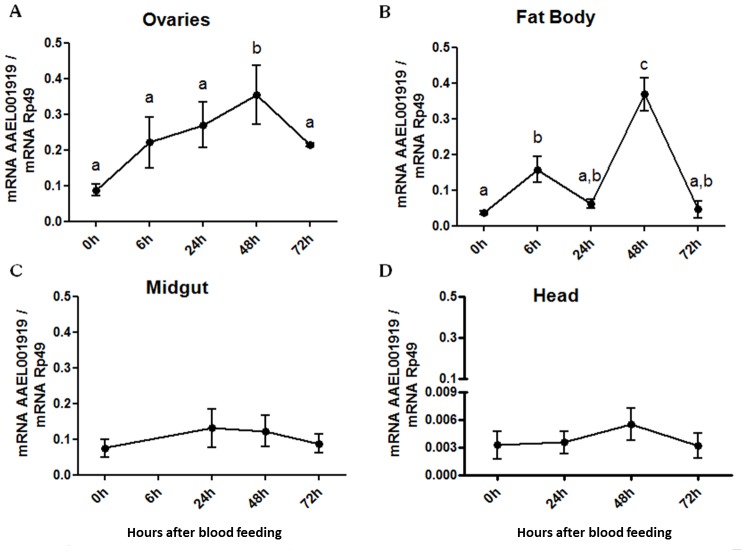
Expression of AAEL001919 after blood feeding. Five- and seven-day old female mosquitoes (20–30 insects) were naturally fed with rabbit blood and dissected 24 h, 48 h and 72 h later. The following tissues were evaluated for AAEL001919 gene expression: (A) Ovaries; (B) Fat body; (C) Midgut and (D) Head. Non-blood fed mosquitoes (0 h) were maintained on 10% sucrose *ad libitum* and were concomitantly dissected at any one of the three time points. Normalized data from five experiments were analyzed by one-way analysis of variance (ANOVA) and by a post-test Tukey's Multiple Comparison Test (a, b, c *p*<0.05). Groups assigned with the same letter (a, b or c) indicate that they do not show statistically significant differences among them.

**Figure 9:**
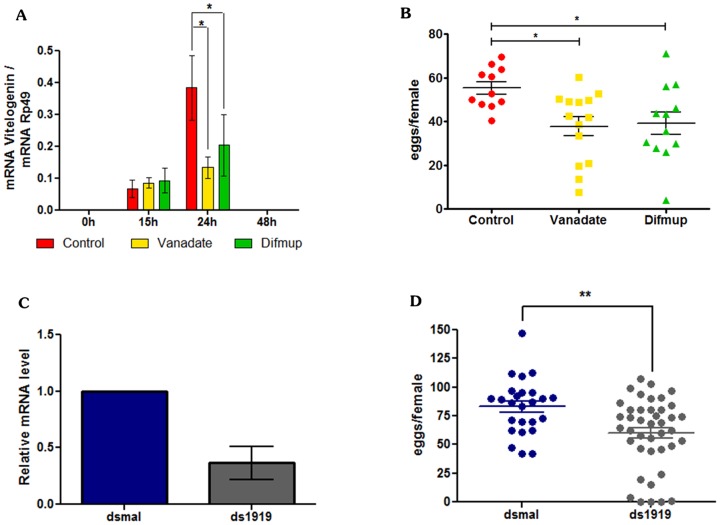
Effect of PTP inhibitors and silencing action of the AAEL001919 gene on egg formation in *Aedes aegypti*. (A) Five- and seven-day old females were artificially fed with rabbit blood supplemented with the PTP inhibitor vanadate or the PTP substrate Difmup. Following a blood meal, fat bodies were dissected from females at the indicated times, and the expression of Vg mRNA was measured by qPCR and normalized against Rp49 gene expression. (B) Females from the experiments shown in A were placed in individual tubes, and the eggs laid were quantified on subsequent days after the blood meal. (C) One- and two-day old females (5 insects) were injected with either 140 ng of AAEL001919 RNAi or dsMal. AAEL001919 expression levels were quantified three days after RNAi injection as shown. (D) Injected mosquitoes from the experiment shown in panel C were naturally blood-fed three days later, and the number of eggs laid following the blood meal was quantified. Normalized data from four experiments were analyzed by One-way ANOVA (panels A and B) and Student's t-test (Panel D) with significance levels set at p<0.05. Groups assigned with the same letter (a, b or c) indicate that they do not show statistically significant difference among them. Different letters in different groups indicate that such groups show significant differences among themselves (Panels A and B). Asterisks indicate (Panel B) indicate a significant difference among groups (student T- test, p<0.05).

Subsequently, we analyzed the expression of AAEL001919 in mosquito tissues. Blood feeding induced a parallel increase in AAEL001919 expression in the ovaries and the fat body, with major peaks at 48 hours in both tissues (Figure 8). Finally, including the classical PTP inhibitor vanadate or the PTP substrate DiFMUP in a blood meal modified the dynamics of Vg synthesis in the mosquito fat body (Figure 9A). It also decreased the total number of eggs laid by females treated in this manner (Figure 9B). This effect on egg formation was mimicked by RNAi silencing of AAEL001919 (Figures 9C and 9D). Taken together, these results indicate the involvement of the PTP gene AAEL001919 in egg formation in *A. aegypti*.

## Discussion

In mammalian cells, the down regulation of insulin pathway is promoted by the activation of either PTEN or PTP1B. PTP1B is classically described as an enzyme involved in the dephosphorylation of phosphotyrosine-containing substrates, especially the insulin receptors in tissues such as liver, muscle and adipocytes. PTP1B usually associates with IR and IRS1 and dephosphorylates these targets. Other classical targets of mammalian PTP1B are the substrates downstream of the leptin receptor. These findings have established PTP1B as a major target for the treatment of diabetes and obesity and have triggered great efforts from many laboratories to develop specific inhibitors of this enzyme [3]. Moreover the combination of trapping mutants with chips containing a collection of cellular peptides has confirmed the prediction of the phosphorylation of the following five high-ranking PTP1B substrates in mammalian cells: PLC-γ1, Gab1, SHP2, EGFR and SHP1 [32], [33]. Furthermore, this strategy has demonstrated, for the first time, that TC-PTP, an enzyme that is closely related to PTP1B, differs functionally from PTP1B and that their substrate specificities overlap only partially. Thus, these findings imply a broader range of functions for PTP1B and PTP1B-like enzymes, indicating that they are not solely involved in the regulation of glucose homeostasis. Several strategies have been employed in the literature to evaluate the role of PTP1B in cell biology [32], [33]. These strategies include overexpression of the enzyme in cultured cells, its knockout in mice, or the physical association of proteins with the active site of the enzyme using substrate-trapping mutants and analysis of the results using mass spectrometry [32]–[35]. Thus, PTP1B is, in fact, a pleiotropic enzyme that acts as a master regulator of several cell-signaling networks [32], [33]. It is possible that vector biology may benefit from such findings by using a similar approach to block PTP1B activity in mosquitoes and, thus, avoid pathogen transmission.

The insulin pathway is involved in diverse aspects of Dipteran biology, including lifespan, immunity and egg formation [29], [30], [31]. As mentioned above, the insulin pathway in mosquitoes acts as an ambivalent modulator of metabolism and immunity [9], [36]–[39]. Several members of the canonical pathways activated by insulin in mosquitoes have been described in recent years, but little is known about negative regulators of this pathway [6], [40]. Such regulators may include PTPs, which are master regulators of tyrosine dephosphorylation. In mosquitos, insulin and insulin-like peptides also modulate the proliferation of immune cells known as hemocytes [39]. The inhibition of these regulators or blocking the expression of proteins under their transcriptional control can potentially provide new targets for molecular modeling and for blocking egg formation and pathogen transmission by mosquitoes [14]. Thus, identifying negative modulators of such pathways is an epidemiologically relevant task. Activation of the PI3K/Akt pathway by down-regulation of a specific *Aedes* PTEN (AaegPTEN6) leads to an increase in oviposition [10]. Furthermore, PTEN overexpression has been shown to extend the mosquito lifespan and increase resistance to *P. falciparum* development [40].

The pharmacological inhibition of PTPs by pervanadate and the silencing of AAEL001919 by RNAi decreased egg production (Figure 9). It is important to mention that pervanadate has been previously shown to affect ecdysone production by mimicking insulin effects on mosquito ovaries [9]. However, the same study showed that pervanadate treatment exhibits a bell-shaped activity curve, whereby either low or high doses of the compound can inhibit ecdysone production [9]. These results suggest that PTPs modulate the phosphorylation states of proteins in this pathway. This effect is different from the effects of PTEN silencing, which ultimately activate the PI3K-Akt branch of the insulin signaling pathway and increase egg production. PTENs are lipid phosphatases whose predominant enzymatic activity appears to be the dephosphorylation of phosphoinositides, ultimately leading to the shutdown of the insulin pathway. This is not the case with PTP1B, whose substrates are largely within but not restricted to components of the insulin pathway [32], [33].

In the present study, we evaluated mosquito PTP structure. We have started with all the genes coding for classical enzymes from this family, but four enzymes were more specifically addressed (Figures 1 to 6). AAEL008528 is involved in transducing signals from the receptor tyrosine kinase Torso. Corkscrew and AAEL008528 are also orthologs of the human gene PTPN11, which encodes the PTP SHP2 protein. Mutations in PTPN11 are related to the development of human diseases known as NOONAN and LEOPARD syndromes. These two syndromes have similar effects but differ in their underlying mechanisms, which result in a gain of function in the case of NOONAN and a loss of function in LEOPARD. The role of this mosquito PTP through differently available phenotypes should be investigated in the futures. Regarding AAEL003108 in *Drosophila*, the vertebrate homologs of PTPN3 (PTPH1) and PTPN4 (MEG1) are expressed in the nervous system, where they are involved in the regulation of axon projections [27], [28]. Despite their presence in mammalian T cells, the roles of PTPN3 and PTN4 are not clear, but their silencing does not affect proper signaling in this model [27], [28]. The *Drosophila* TCPTP/PTP1B ortholog dmPTP61F codes for two splicing variants that are differentially localized in the cell. The isoform localized in the endoplasmic reticulum is shown to be able to regulate insulin signaling in vivo [29]. Mutants lacking both PTP61F variants display a decrease both in mean life span and in female fecundity [29]. Furthermore, it has been shown previously that PTP61F dephosphorylates the *Drosophila* insulin receptor in S2 cells in vitro and acts as a negative regulator of the *Drosophila* JAK/STAT pathway [30], [31].

To ensure that our observations can be interpreted as the enzymatic behavior of PTPs, appropriate controls were performed including enzyme activity initially using pNPP, a generic substrate for phosphatases [24], [39]–[46]. Assays were performed in the presence or absence of micromolar levels of orthovanadate, a classical PTP inhibitor. Under these conditions, this inhibitor always blocked 40–90% of the overall activity, either with pNPP or a specific peptide substrate for PTP1B (Figure S1). To reveal specific effects of PTP1B on mosquito biology, particularly in events during the vitellogenic phase, we studied egg laying in the absence or presence of vanadate and detected a vanadate-induced decrease in egg formation. Surprisingly, even at high concentrations of the inhibitor, only 15–25% of the PTP activity was abolished in the presence of this molecule. Despite this, no structurally or conformationally relevant differences exist between PTP1B in human and *A. aegypti*, an observation also supported by the structural approach conducted at the beginning of this study (Figures 1 to 4, Tables 1 and 2). This inhibitor-susceptibility difference may open paths for the design of specific inhibitors for different groups of organisms. Furthermore, these results reinforce the idea that PTPs play a key role in the oogenesis of *A. aegypti*.

Analysis of tyrosine phosphoproteomics in *Drosophila* S2 or the knockdown of PTP61F in *Drosophila* Schneider cells led to the identification of several dPTP61F substrates [34]. Comparison of those results with another study addressing the role of Albeson tyrosine kinase (Abl) and Abl interacting protein (Abi) in *Drosophila* oogenesis revealed Abi/Abl complex as a direct target of dPTP61F [34], [47]. Abl phosphorylates Abi, which is also a direct target of dPTP61F-mediated dephosphorylation. Thus, the silencing of dPTP61F resulted in an increase of Abl phosphorylation and activity and an increase in Abi activity. Abi functions as a substrate adaptor protein for Abl and a core member of the SCAR/WAVE complex, relaying signals from Rac to Arp2/3 and regulating actin dynamics. Tyrosine phosphorylation of SCAR/WAVE by Abl is required for actin polymerization [47]. Thus, the silencing of AAEL001919 in *Aedes* may lead to an impairment of cytoskeleton dynamics associated with egg formation that ultimately blocked egg production. Regarding the effect of AAEL001919 on Vg synthesis (Figure 9) we speculate that such effect may occur through the up regulation of enzyme expression (Figure 8B). RT-PCR data obtained here were also analyzed in comparison with the RNA-seq results published by another group [48]. Such results regarding the expression of PTP genes described in the present study match each other. Thus the analysis of all PTP genes reported here indicate a similar increase on AAEL001919 after a blood meal but the exact point in time when this occurs is difficult to define due to different approaches used by both studies. Nevertheless, AAEL001919 as reported by Bonizzoni et al. 2011 is the major PTP gene affected by a blood meal [48].

In conclusion the analysis of *Aedes* classical PTPs reported here led to the identification of a protein encoded by the AAEL001919 gene and showing great structural similarity to human and *Drosophila* PTP1B. This protein may be a major negative regulator of the insulin pathway in mosquitoes, but its direct targets remain unknown, and their identity was not addressed in the present investigation. Insulin receptors (IRs) usually present high conservation, especially in the kinase catalytic domain that is essential for their activation, but the PTP1B target region (that contains tyrosine phosphorylation sites) is considered the most variable part of these receptors. The high level of similarity in PTP1B is interesting because the IR variable region would be expected to guide selective pressure on PTP1B for the purpose of concomitant changes [41]. The differences in inhibitor susceptibility among PTPs may allow drug design through a strategy that generates a drug that only affects the insect phosphatase or its substrates, such that it is inactive in mammals. In *D. melanogaster*, the ortholog of PTP1B, the dmPTP61F gene, generates two products that differ only by a small C-terminal sequence and that contain the same membrane-binding domain. This difference allows the product containing this specific domain to become anchored to the endoplasmic reticulum, whilst the product that lacks the specific domain exhibits nuclear localization [41], [42]. Interestingly, in mammals, the membrane-bound PTP1B has been reported to have an inhibitory effect on the insulin signaling pathway, leading to insulin resistance, and has also been shown to be capable of causing an increase in blood glucose [41], [42]. In contrast, the PTP1B protein with the truncated membrane-binding C-terminal domain can still lead to insulin resistance but is unable to activate SREBP-1 [42]–[44]. In conclusion, while it is likely that AAEL001919 regulates glucose metabolism and immunity in mosquitoes, the present study clearly demonstrates its involvement in egg formation in *Aedes aegypti* females. Furthermore, this enzyme demonstrates potential use as a target for designing specific inhibitors that block mosquito egg development and vector-mediated disease transmission.

## Materials and Methods

### Reagents

Tris, glycine, acrylamide, bis-acrylamide, TEMED, DMSO, DTT, bovine serum albumin, sodium vanadate, okadaic acid, Folin reagent and pNPP were obtained from Sigma-Aldrich Company (St. Louis, MO, USA). Prestained Full-Range Rainbow molecular weight standards were obtained from Amersham Biosciences (Buckinghamshire, England). Ethanol, Triton X-100 and DMSO were obtained from Merck (Darmstadt, Germany).

### Ethics statement

All animal care and experimental protocols were conducted following the guidelines of the institutional care and use committee (Committee for Evaluation of Animal Use for Research from the Federal University of Rio de Janeiro, CAUAP-UFRJ) and the NIH Guide for the Care and Use of Laboratory Animals (ISBN 0-309-05377-3). The protocols were approved by CAUAP-UFRJ under registry IBQM067-05/16. Technicians dedicated to the animal facility at the Institute of Medical Biochemistry (UFRJ) carried out all aspects related to rabbit husbandry under strict guidelines to insure careful and consistent handling of the animals.

### Mosquito rearing and blood meals

*Aedes aegypti* (Liverpool Black Eye strain) were raised in an insectary at the Federal University of Rio de Janeiro, Brazil, under a 12-h light/dark cycle at 28°C and 60–80% relative humidity. Larvae were fed with dog food (Pedigree Junior), and adults were maintained in a cage and given a solution of 10% sucrose *ad libitum*. Five- to seven-day old females were used in the experiments. Mosquitoes were naturally fed on rabbits' ear veins or were artificially fed with heparinized rabbit blood. Artificial feeding was performed using water-jacketed artificial feeders maintained at 37°C and sealed with parafilm membranes. Whenever indicated blood was supplemented with PTP inhibitors.

### Phosphatase assay using *para*-Nitrophenylphosphate (*p*NPP)

The tissues were homogenized in buffer containing 20 mM of sodium acetate (pH 4.0). The protein concentration of each extract was determined by the Lowry method. The homogenates were used to perform the phosphatase assays, using 0.5–3 µg of protein, depending on the tissue, and using *p*-nitrophenylphosphate (pNPP) as substrate. Enzyme activity was measured in the presence or absence of 100 µM sodium orthovanadate, used as tyrosine phosphatase inhibitor, to determine the specific activities of PTPs. The reactions were conducted at 37°C for 60 minutes and were stopped by adding 2 N NaOH. In some experiments, we included 0.1 mM of PTP 1B substrate II (Glu-Leu-Glu-Phe-pTyr-Met-Asp-Tyr-Asp-Tyr-Glu) (Calbiochem) in addition to pNPP. Further conditions were as described previously [24], [49].

### *In silico* analysis of *Aedes* PTPs

Identification and classification of tyrosine phosphatases (PTPs) in mosquitoes were accomplished by searching conserved domains from Pfam. The search for conserved domains (CDs) employed FAT [50], a program developed by our group. It is a HMMER filter and blast manager. First, proteins predicted (version 1.3) by Vector-Base in the *A. aegypti* genome were downloaded (https://www.vectorbase.org/download/aedes-aegypti-liverpoolpeptidesaaegl13fagz). Using FAT, the sequences were filtered using characteristic domains of PTP families (Class I soluble classical PTPs used Y_phosphatase domain PF00102). The sequences obtained were compared using blast with databases such as nr, Swiss-prot and a custom human tyrosine phosphatases database. Lastly, hmmscan searched for other conserved domains in these previously filtered proteins. While this manuscript was in preparation, we became aware of another publication that described a specific sequence-based method for the automatic classification of PTPs present in 65 genomes, including *A. aegypti* [21]. The same classical PTPs described in that study were confirmed by the strategy described here.

### Analysis of PTP sequences, protein models and active-site modeling

*In silico* approaches were used to ascertain the function and the evolutionary homologs of *Aedes PTPs*. Because proteins with high sequence and structural similarity may possess the same biological functions, the *in silico* approach was used to find the closest homologs of PTPs in an effort to obtain homology gene models [50]. Sequences were used to search the homologue sequence space for related proteins using the NCBI BLAST server [50]. A BLOSUM62 matrix was used for coring of the sequences, with an expected threshold of 10 and a sampling size of 3 amino-acids at a time. The non-redundant protein database was used for searching the sequence space without filtering out low-complexity regions. To identify the homologues with known protein structures, the PSI-BLAST tool was used against the freely available Protein Data Bank. PSI-BLAST allowed for better sensitivity and enabled us to find the closest five structures, which could be used in homology modeling. These five sequences were used for a multiple sequence alignment using the MULTALIGN server [51]–[53]. In several instances, the *Aedes* PTP and the homologous PTP sequences had gaps, so the modeler could not be effectively used for homology modeling. In these cases, the automated Protein Homology/analogy Recognition Engine was used to find the respective homology models [54]. The reliability of the models was checked via submission to the WHAT IF server [55], [56]. The protein structures were visualized and superimposed using PyMOL software (DeLano Scientific LLC). The various domains in the sequences were identified using a combination of online servers, including the Conserved residue Function Prediction Server [56] and the Conserved domain Database at the NCBI [57]. The active site of the Protein-Tyrosine-Phosphatase domain was ascertained using the Catalytic Site Atlas [58] as well as the siteFinder web-based tool [59]. The ten motifs that define the PTP domain were identified and used, as defined in the PTP database [60]. Secondary-structure prediction for the sequence corresponding to the PTP domain was obtained from the Psipred server [61]. Sequences of human and fly (*Drosophila melanogaster*) PTPs were obtained from the non-redundant Protein Database at NCBI and used for construction of dendrograms using the CLUSTALW web server and the TREEVIEW software package.

### RNA extraction and qPCR analysis

RNA was extracted from each sample using TRIzol reagent (Life Technologies). RNA quantification was accomplished using a NanoDrop-3300 (Thermo Scientific) device before cDNA synthesis was performed. Briefly, 1 µg of RNA was treated with RNase-free DNase (Fermentas) to avoid genomic DNA contamination. Subsequently, cDNA synthesis was performed using the High Capacity cDNA Reverse Transcription kit (Applied Biosystems), according to the manufacturer's protocol. Transcript levels were determined by Real Time PCR using a SYBR-Green-based method. The quantitative PCR assays were employed a StepOnePlus Real Time PCR System (Applied Biosystems) using PowerSYBR Green PCR Master Mix (Applied Biosystems). Gene expressions, as evaluated by mRNA levels, were calculated by normalization to the levels of Ribosomal protein 49 (*rp49*) mRNA (accession number AAT45939), which served as the endogenous control in each individual sample, and taking into consideration the respective efficiency of each pair of primers. The comparative ΔΔCt method was then used to calculate relative gene expression levels, and all standard errors were calculated based on ΔCt, as described in the Applied Biosystems User Bulletin #2 (http://www3.appliedbiosystems.com/cms/groups/mcb_support/documents/generaldocuments/cms_040980.pdf). The primers used presented an efficiency of at least 95%. The primer pairs corresponding to the analyzed genes were as follows: AAEL001919 (fwd 5′ GATTGGCG AAGAGCACAAATTG, rvs 5′ TAATCGGAACGTCCTTTTGC 3′), AAEL005492 (fwd 5′ GTGATAGTAATGACCACTCG 3′, rvs 5′ ACCTGATAGCATCCATATTC 3′), AAEL008528 (fwd 5′ GGGTCCAATTTGTGTCCAC 3′, rvs 5′ GATCTCGCAGTCCAGACC 3′), AAEL003108 (fwd 5′ATGGTACAACAGGAAAGCAG 3′, rvs 5′GATGGAGAATCCTTCAGACA 3′), AAEL010234 (fwd 5′ TAGATTTACGGTGGCGGAC 3′, rvs 5′ GCTTGCGTCTGGTTCTTTTC 3′), AAEL010914 (fwd 5′ TGGCAGTATGGTGGATAGC 3′, rvs 5′ CAACCGTTCAACCTCCTTG 3′), AAEL001046(fwd 5′AAGCAACCGAACTTTGTTGG 3′, rvs 5′CCCGTTGAGTTGGTCT GATT 3′), Rp49(fwd 5′TCAACCCCCGTTCGAACA 3′, rvs 5′CCGTAACCGATGTTTGGC3′), Vg(fwd 5′ TGAATTTGTCACCCCCGATC 3′, rvs 5′ TTCACGCTTGACACATTCCTG 3′).

### dsRNA synthesis and injections

Double-stranded RNA was synthesized using a MEGAscript RNAi kit (Ambion, Austin, TX, USA), according to manufacturer's instructions. 138 nL of 3 µg/µL dsRNA solution re-suspended in water was injected into the thorax of cold-anesthetized 1-to-2-day-old female mosquitoes. dsRNA injections employed a nanoject II nanoliter injector (Drummond Scientific). Primers' sequences used for template amplification for dsRNA synthesis were as follows: RNAi1919F 5′ TAATACGACTCACTATAGGGTGCCACCGTTACCTAAGGAC 3′ and RNAi1919R 5′ TAATACGACTCACTATAGGGCTGGGCTAGACACTGCTTCC 3′. These primers contained a T7 polymerase binding sequence, required for dsRNA synthesis. As a control, we used maltose-binding protein (mal) from *Escherichia coli* dsRNA (dsmal). This sequence was inserted into a pBlueScript KS+ (Stratagene) and was amplified using the T7 minimal promoter primer (5′ TAATACGACTCACTATAGGG 3′) for template generation and dsRNA synthesis.

### Evaluation of the Role of PTP on Egg Oviposition

In some experiments mosquitoes were fed with 10% sucrose enriched either with the 0.1 mM of the PTP inhibitor sodium orthovanadate or the PTP substrate DifMUP. In these experiments cages with 10–15 mosquitoes were fed with blood in the presence or absence of the mentioned PTP modulators and 72 hrs later the number of laid eggs was evaluated for the whole cage. At least three different cages were used in each experiment. Therefore each plotted point on the graphics (Figure 9B) is the number of eggs in a single cage divided by the number of females present on that cage. RNAi silencing of AAEL001919 was also used as a strategy to block PTP activity. In each of these experiments a total of 30 mosquitoes where either injected with dsMal or ds1919 as indicated in figure legend. Following injection groups of 2 mosquitoes were kept in separate in Falcon tubes in order to decrease mortality. The plotted results indicate the total number of eggs laid in each Falcon divided by 2 female mosquitoes.

### Statistical Analysis

All experiments were performed at least in triplicate. The results are presented as the means and standard errors of the mean. Normalized data were analyzed by One-way ANOVA or Student's t-test using GraphPad Prism software.

## Supporting Information

Figure S1 **Effect of a tyrosine phosphorylated peptide on PTP activity towards pNPP on different tissues of** ***A. aegypti*** **.** Tissues dissected from 10% sucrose-fed females (were homogenized and enzyme activity towards pNPP was assayed in the presence or absence of 0.1 mM of PTP 1B substrate II. After 30 minutes at 37°C, the reactions were halted by adding 1∶8,5 volumes of 2 M NaOH. Results represent the standard two determinations in triplicate and mean deviation.
(TIF)
