# Supplementary figures and images for: Molecular Analysis of Aedes aegypti Classical Protein Tyrosine Phosphatases Uncovers an Ortholog of Mammalian PTP-1B Implicated in the Control of Egg Production in Mosquitoes
Source: PLoS One. 2014 Aug 19;9(8):e104878. doi: 10.1371/journal.pone.0104878 (PMC4138107; doi:10.1371/journal.pone.0104878)

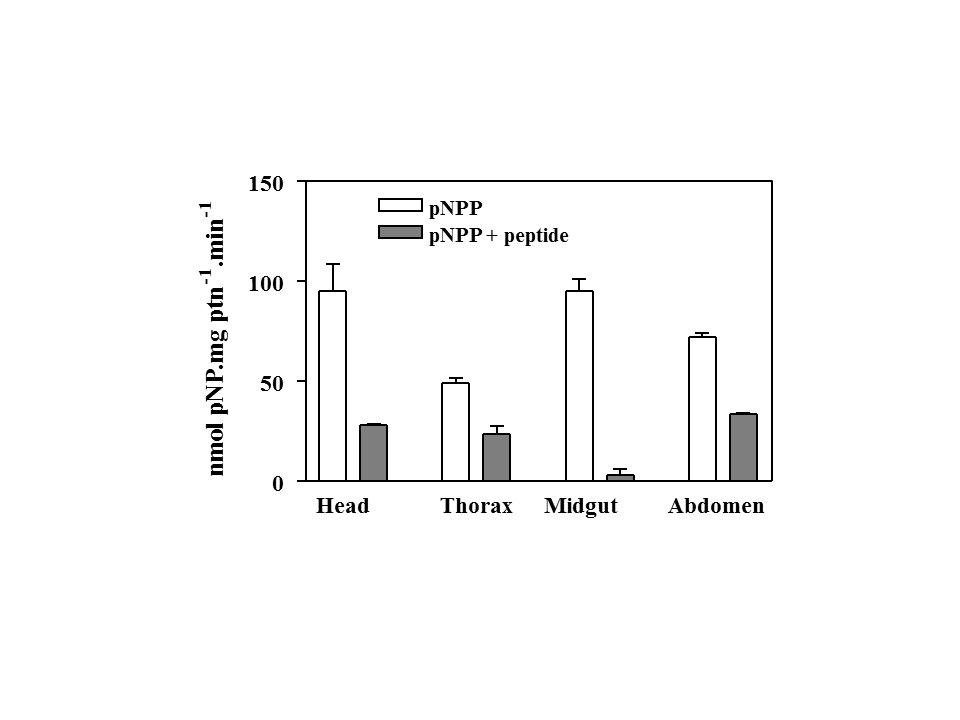

Supplement: Figure S1 — Effect of a tyrosine phosphorylated peptide on PTP activity towards pNPP on different tissues of A. aegypti . Tissues dissected from 10% sucrose-fed females (were homogenized and enzyme activity towards pNPP was assayed in the presence or absence of 0.1 mM of PTP 1B substrate II. After 30 minutes at 37°C, the reactions were halted by adding 1∶8,5 volumes of 2 M NaOH. Results represent the standard two determinations in triplicate and mean deviation. (TIF) [file pone.0104878.s001.tif]
